# Proteomic profiles of *Lissachatina* (Heterobranchia) and *Pomacea* (Caenogastropoda) snails infected with *Angiostrongylus cantonensis* using 4D label-free quantitative analysis

**DOI:** 10.1371/journal.pntd.0013812

**Published:** 2025-12-08

**Authors:** Peter S. Andrus, Li-min Yang, Qing-chi Han, Zhi-heng Qi, Zhi-ying Hou, Xiao-nen Wu, Si-yuan Liu, Kun Wang, Jun-hu Chen, Robbie Rae, Christopher M. Wade, Yun-hai Guo, Xiao-nong Zhou

**Affiliations:** 1 School of Global Health, Chinese Center for Tropical Diseases Research, Shanghai Jiao Tong University School of Medicine, Shanghai, China; 2 National Key Laboratory of Intelligent Tracking and Forecasting for Infectious Diseases, National Institute of Parasitic Diseases, Chinese Center for Disease Control and Prevention; Chinese Center for Tropical Diseases Research, Key Laboratory on Parasite and Vector Biology, Ministry of Health, WHO Collaborating Centre for Tropical Diseases, National Center for International Research on Tropical Diseases, Ministry of Science and Technology, Shanghai, China; 3 Hainan Tropical Diseases Research Center (Hainan Sub-Center, Chinese Center for Tropical Diseases Research), Hainan, China; 4 Hangzhou Medical College, Hangzhou, China; 5 School of Biological and Environmental Sciences, Liverpool John Moores University, Liverpool, United Kingdom; 6 School of Life Sciences, University of Nottingham, Nottingham, United Kingdom; University of Passo Fundo: Universidade de Passo Fundo, BRAZIL

## Abstract

*Angiostrongylus cantonensis*, the causative agent of human eosinophilic meningitis, utilizes terrestrial and freshwater gastropods as intermediate hosts. However, the molecular mechanisms underlying these host-parasite interactions remain unclear. We applied four-dimensional label-free quantitative (4D-LFQ) proteomics to examine proteomic alterations in infected versus uninfected specimens of two intermediate snail hosts, *Lissachatina fulica* and *Pomacea canaliculata*. Differentially expressed proteins (DEPs) were identified, followed by Gene Ontology (GO) enrichment and Kyoto Encyclopedia of Genes and Genomes (KEGG) pathway analyses. In infected *Lissachatina*, 36 proteins were upregulated and 104 downregulated, while in infected *Pomacea*, 94 were upregulated and 364 downregulated. GO analysis revealed 111 enriched terms linked to 71 DEPs in *Lissachatina* and 484 terms associated with 389 DEPs in *Pomacea*. KEGG pathway enrichment (Level 3) showed predominant downregulation, including 12 of 20 pathways in *Lissachatina* and 18 of 20 in *Pomacea*. Both species shared downregulation in essential pathways: ribosome, proteasome, aminoacyl-tRNA biosynthesis (genetic information processing); glycolysis/gluconeogenesis, pyruvate metabolism, sulfur metabolism (metabolic); and phagosome formation and endocytosis (immune-related). Protein–protein association (PPA) analysis identified conserved hub proteins, Tr-type G domain and T-complex chaperonins, indicating coordinated disruption of translational and proteostatic processes in both groups. Our findings suggest that *A. cantonensis* can modulate host immunity and metabolism, suppressing key protective responses in both gastropod hosts. This proteomic data may serve as a foundation for discovering biomarkers and designing interventions to disrupt the parasite’s life cycle.

## Author summary

*Angiostrongylus cantonensis* is a parasitic nematode that causes eosinophilic meningitis in humans and relies on a broad range of gastropod species to complete its life cycle. However, little is known about how this parasite interacts with its intermediate gastropod hosts at the molecular level during development. In this study, we investigated two key intermediate hosts, *Lissachatina fulica* and *Pomacea canaliculata*, to better understand how *A. cantonensis* infection alters host biology. Using advanced four-dimensional label-free quantitative (4D-LFQ) proteomics, we found that infection with *A. cantonensis* causes widespread downregulation of proteins involved in metabolism, gene expression, and immune function in both snail species. In particular, pathways related to energy production, protein synthesis, and pathogen defence were significantly suppressed. These findings suggest that *A. cantonensis* could be actively reprogramming the cellular environment of its hosts, weakening their physiological and immune responses to facilitate its own survival and development. This study provides new insights into the molecular basis of host manipulation in gastropods and offers potential targets for disrupting parasite transmission.

## 1. Introduction

Zoonotic diseases are responsible for over 60% of all human infections and 75% of emerging infectious diseases globally [1]. Parasitic infections impose a major global health burden, impacting approximately 25–30% of the population (~3.5 billion people). Among these, helminths (e.g., cestodes, trematodes, and nematodes) infect over two billion individuals worldwide [2,3]. Despite their medical importance, the evolutionary origins and molecular mechanisms underlying helminthic parasitism remain poorly understood, with parasitism having evolved independently multiple times within different nematode lineages [4]. Understanding these processes is critical for developing novel strategies to control parasitic diseases. However, effective control is challenging due to the parasites’ complex life cycles, often involving multiple hosts and animal reservoirs. For example, *Angiostrongylus* is a genus of parasitic nematodes that uses gastropods as intermediate hosts and rats as definitive hosts. Humans can become accidental hosts through the ingestion of third-stage larvae (L3), typically via contaminated water, raw vegetables, or undercooked gastropods and other paratenic hosts [5]. Human angiostrongyliasis is recognized as the leading cause of eosinophilic meningitis worldwide, and was officially listed as an emerging food-borne parasitic disease by the World Health Organization in 2002, and as an emerging infectious disease by the Chinese Ministry of Health in 2004 [6]. However, despite it widely being regarded as a neglected disease by researchers [7,8], it is not currently listed among the WHO’s Neglected Tropical Diseases (NTDs), or Neglected Parasitic Zoonoses (NPZs). Human angiostrongyliasis is primarily caused by *Angiostrongylus cantonensis*, which can result in severe, debilitating, or fatal neurological conditions such as eosinophilic meningitis and encephalitis [9]. The global distribution of *A. cantonensis* is concentrated in Southeast Asia (e.g., China, Thailand, Malaysia) and the Pacific Islands (e.g., New Caledonia, Fiji, Cook Islands) [10]. However, due to climate change, globalization, and the spread of invasive gastropod species, its range has expanded to Africa, Australia, the Americas, and Europe [7]. Globally, over 7,000 cases of human angiostrongyliasis have been reported [11], although the true burden is likely much higher due to underdiagnosis and underreporting, particularly in low-resource settings [12].

Angiostrongyliasis is an increasingly important public health concern, particularly as globalization accelerates the spread of *A. cantonensis* beyond its traditional endemic areas. In recent years, the number of reported human cases has grown rapidly, with the majority of infections occurring in South and South East Asia [7,13]. This global expansion is not only driven by human movement and trade but also by the widespread distribution of invasive intermediate host snails, heightening the risk of imported infections worldwide. *Angiostrongylus cantonensis* can infect a broad range of terrestrial and freshwater gastropod species, with at least 199 species being able to act as compatible intermediate hosts [14]. However, over the past decade, there has been increasing interest in the two primary intermediate snail hosts of *A. cantonensis*, *Lissachatina fulica* (Bowdich, 1822) and *Pomacea canaliculata* (Lamarck, 1819) as they are frequently found infected, are both highly invasive and widely consumed [15,16]. Both species have spread across Asia, the Americas, Africa, and the Pacific, contributing to the global expansion of *A. cantonensis* and the increased incidence of human angiostrongyliasis [7]. A meta-analysis of *A. cantonensis* infection prevalence in *Lissachatina* and *Pomacea* snails across China found *Lissachatina* snails had a higher prevalence (21.5%) than *Pomacea* snails (7.6%) [16]. Both *Lissachatina* and *Pomacea* snails often inhabit urban environments where contact with infected rodent populations is more frequent, resulting in their significant role in sustaining local transmission cycles of *A. cantonensis*.

Infection occurs when snails ingest or come into contact with first-stage larvae (L1) excreted in the feces of infected rats. The larvae penetrate the snail’s epithelium and migrate into internal tissues, undergoing two molts to become infective third-stage larvae (L3). The larvae spread widely throughout the snail’s body, with hemolymph serving as a key immunological barrier against infection. However, like many parasitic nematodes, *A. cantonensis* is hypothesized to have immunomodulatory properties, potentially altering the host immune landscape to facilitate its survival [17,18]. For example, *A. cantonensis* secretes galectin-1, which interacts with mammalian host proteins such as Annexin A2 to induce apoptosis in macrophage-like cells via the JNK signaling pathway [19]. This mechanism not only depletes immune cell populations but also disrupts critical signaling pathways required for mounting effective immune responses against the parasite. Similarly, in gastropod hosts, this may involve suppression of hemocyte activation, and the downregulation of antimicrobial peptide production, or inhibition of key immune signaling pathways [20–23]. However, the specific molecular interactions and genetic pathways between *A. cantonensis* and its gastropod hosts remain largely unknown. Therefore, gaining insight into these processes is essential for elucidating how the parasite develops and persists within susceptible intermediate gastropod hosts.

Proteomics provides a powerful tool for exploring these interactions by identifying differentially expressed proteins (DEPs) during infection. To date, few studies have examined proteomic responses in gastropods infected with *A. cantonensis* [24,25], with most proteomic research focusing on developmental stages of the parasite itself [26–28] or the differences in protein expression of the parasite in the definitive host [29,30]. Recent advances in mass spectrometry, particularly 4D-label free quantification (4D-LFQ), offer enhanced sensitivity and resolution, which is ideal for analyzing complex, low-abundance samples like snail hemolymph [31]. This method offers superior sensitivity, dynamic range, and the ability to detect low-abundance proteins, making it especially suitable for limited, or complex biological samples (i.e., snail hemolymph). In this study, we used 4D-LFQ proteomics to characterize immune and metabolic responses in the hemolymph of *Lissachatina fulica* and *Pomacea canaliculata* following infection with *A. cantonensis*. DEPs were identified and functionally analyzed through Gene Ontology (GO) and Kyoto Encyclopedia of Genes and Genomes (KEGG) enrichment. Finally, protein–protein association (PPA) network analysis was applied to map functional connections among DEPs and pinpoint central hub proteins that may coordinate host stress and immune responses during infection [32]. Our aim was to investigate infection-induced proteomic changes to better understand how the parasite modulates host pathways to support its development and transmission.

## 2. Results

**Fig 1:**
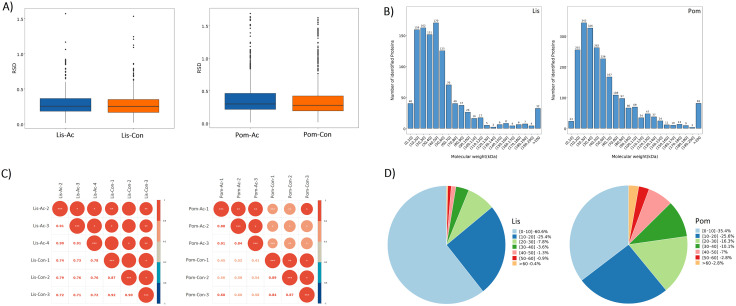
Quality assessment and clustering of trusted protein expression data in *A. cantonensis*-infected and uninfected *Lissachatina* (Lis) and *Pomacea* (Pom) groups. (A) Boxplots showing the distribution of relative standard deviation values across biological replicates for *Lissachatina* and *Pomacea* samples. (B) Histograms showing the number of identified proteins across different molecular weight (kDa) ranges of the *Lissachatina* and *Pomacea* samples. (C) Sample correlation plots of the *A. cantonensis*-infected and uninfected *Lissachatina* and *Pomacea* groups. (D) Pie charts showing the distribution of sequence coverage among identified proteins. The percentages represent the proportion of proteins within each sequence coverage interval.

A spectral analysis (FDR < 0.01) identified a total of 2766 peptides and 1087 proteins for the *Lissachatina* dataset, while a higher number of peptides (15,819) and proteins (2211) were identified in the *Pomacea* dataset. To evaluate the reproducibility of biological replicates, the relative standard deviation of trusted protein expression was assessed across infected and control groups (**Fig 1A**). The relative standard deviation values were comparable between replicates, indicating good consistency for both the *Lissachatina* and *Pomacea* groups. Next, the molecular weight distribution of identified proteins was measured, with the majority of proteins in both snail groups ranging between 10–50 kDa, with a notable concentration in the 10–30 kDa range (**Fig 1B**). In the *Lissachatina* group, 170 proteins were found in the 20–30 kDa range, while in the Pomacea group, 343 proteins were observed in the 10–20 kDa range. Next, the raw proteomic datasets were retrieved from the database, and the proteins were filtered based on stringent criteria to ensure reliability. Only proteins with at least one unique peptide and an expression value detected in ≥50% of samples within at least one experimental group were retained for further analysis. To address missing values, proteins with ≤50% missing data had their values imputed using the mean expression level of the corresponding sample group. To normalize the data and reduce technical variability, median normalization was applied, followed by log2 transformation to stabilize variance and facilitate interpretation of protein abundance changes. The processed dataset, comprising trusted proteins, was then subjected to statistical evaluation and visual representation to confirm data integrity. A sample correlation plot analysis was performed on the expression profiles of the trusted proteins to assess sample clustering and variability for each of the experimental groups (**Fig 1C**). The sample correlation plot showed the A. cantonensis-infected groups (Lis-Ac and Pom-Ac) had a higher correlation coefficient between one another, than compared to the uninfected groups (Lis-Con and Pom-Con) for both the *Lissachatina* and *Pomacea* groups.

Next, sequence coverage analysis showed that 60.6% of *Lissachatina* proteins had 0–10% coverage and 25.4% had 10–20% coverage (**Fig 1D**). In contrast, *Pomacea* proteins exhibited a broader coverage distribution, with 35.4% of proteins in the 0–10% range and 25.6% between 10–20%. Notably, *Pomacea* had a higher proportion of proteins with coverage above 20%, including 2.8% exceeding 60%, compared with only 0.4% in *Lissachatina*. This broader coverage indicates more complete peptide representation and reflects the availability of a well-annotated reference proteome for *P. canaliculata*, whereas *L. fulica* relies on homologous annotation from related gastropods. For more information on the reproducibility of the biological replicates for each group see **S1A Fig**. In brief, a PCA plot and hierarchical clustering tree based on Euclidean distances was performed to assess the relationships between sample groups (**S1A** **and** **S1B Fig**). Both the PCA plot and hierarchical tree showed the *A. cantonensis*-infected samples were separate from the uninfected samples for both the *Lissachatina* and *Pomacea* groups. Next, boxplots and density plots were generated to examine the distribution of normalized protein expression values, and to visualize the probability distribution of trusted protein expression values (**S1C** **and** **S1D Fig**). The spread and symmetry of the boxplots, and the overlapping density curves of the density plots demonstrated consistent expression patterns among biological replicates.

### 2.1 Differentially expressed proteins (DEPs)

**Fig 2:**
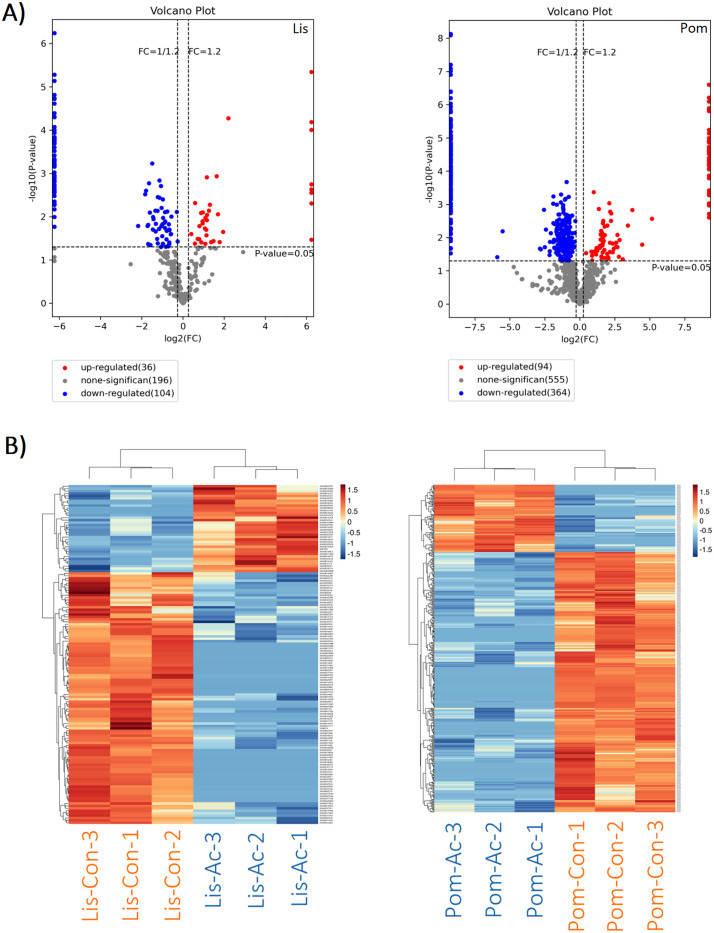
Overview of differentially expressed proteins (DEPs) in *A. cantonensis*-infected samples when compared to uninfected control samples for both the *Lissachatina* (Lis) and *Pomacea* (Pom) groups. (A) Volcano plots illustrating differential protein expression using Fold Change (log₂) values, and p-values (-log₁₀). Red dots represent up-regulated proteins, blue dots represent down-regulated proteins, and gray dots represent non-significant proteins. Vertical dashed lines indicate the fold change thresholds (Fold Change = 1.2 and Fold Change = 1/1.2, or 0.833), and the horizontal dashed line indicates the p-value cutoff (p = 0.05). (B) Hierarchical cluster heat maps of DEPs. Heat maps were generated based on protein expression levels using unsupervised hierarchical clustering. Red indicates proteins with high expression levels, and blue indicates proteins with low expression levels. Each row represents a DEP, and each column represents a sample.

Differential protein screening was performed using the trusted protein dataset. Fold change (log₂ [Fold change]) was calculated as the mean expression value of the experimental group (*A. cantonensis*-infected) minus that of the control group (uninfected). A Student’s t-test was used to calculate p-values for statistical significance. The overall distribution of the DEPs was visualized using volcano plots (**Fig 2A**). In the *Lissachatina* group, 36 proteins were up-regulated (≥1.2 FC, P-value <0.05) and 104 proteins were down-regulated (≤0.83 FC, P-value <0.05) in the *A. cantonensis*-infected group when compared to the uninfected group (**Fig 2A**). In the *Pomacea* group, there was a higher amount of both up-regulated (94) and down-regulated (364) proteins in the *A. cantonensis*-infected group compared to the uninfected group (**Fig 2A**). Next, to further evaluate the expression profiles of the DEPs, an unsupervised hierarchical cluster heatmap analysis was conducted (**Fig 2B**). In both the *Lissachatina* and *Pomacea* datasets, samples grouped according to their infection status, indicating high internal consistency within each group. Proteins exhibiting similar expression patterns were also grouped together, highlighting patterns of co-expression. The clear separation of infected and uninfected groups demonstrates the robustness of the DEP analysis. Lastly, a correlation analysis was conducted on the top 50 DEPs (ranked by p-value; **S2 Fig**). Pearson correlation coefficients were calculated to assess the similarity of expression patterns between proteins, with coefficients closer to 1 indicating stronger positive correlations. Both groups exhibited a predominance of positively correlated protein pairs, with the *Pomacea* group showing more defined clusters of highly co-expressed proteins (**S2 Fig**).

### 2.2 Functional classification and enrichment of DEPs

**Fig 3:**
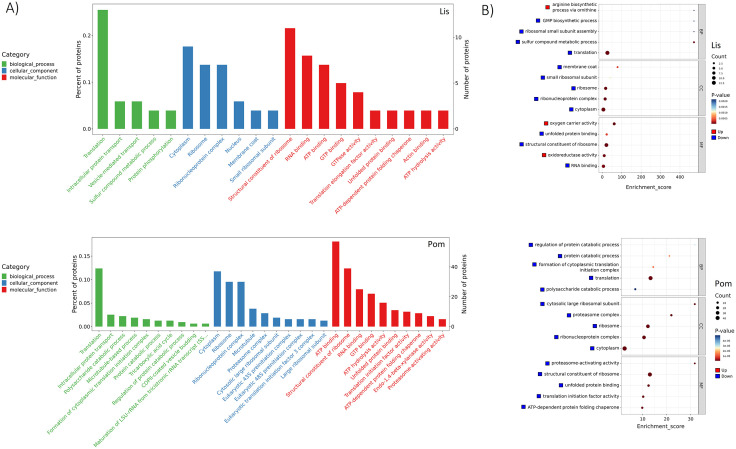
Gene Ontology (GO) functional enrichment analysis of differentially expressed proteins (DEPs) in infected *Lissachatina* (Lis) and *Pomacea* (Pom) groups. (A) Bar plots showing the total number of Level 2 Gene Ontology (GO) annotated DEPs, categorized into Biological Process, Cellular Component, and Molecular Function. (B) Bubble chart of the top five significantly enriched GO terms per ontology category. Bubble size indicates the number of associated proteins, while color intensity represents statistical significance (red = lower adjusted p-value). Only GO terms with more than one protein were included. Enrichment was assessed via hypergeometric testing with Benjamini–Hochberg correction, and results are shown as adjusted p-values and enrichment scores. Note: Full details of GO terms can be found in S1 Dataset.

After identifying the DEPs, Gene Ontology (GO) and Kyoto Encyclopedia of Genes and Genomes (KEGG) enrichment analyses were conducted to reveal significant functional associations of the DEPs, and to better understand the biological roles and pathways involved. These analyses aimed to identify which biological processes, cellular components, and molecular functions were significantly over-represented among the DEPs. To highlight the most relevant functions, the top 30 GO terms were selected based on the number of associated differential proteins (List Hits >1) across the three GO categories: biological process (BP), cellular component (CC), and molecular function (MF). When looking at the total GO terms, we found the *Lissachatina* group had 111 GO terms (BP: 31, CC: 32, MF: 48) made up of 71 DEPs (**Fig 3A**). Of the 71 DEPs, 20 were upregulated and 51 were downregulated. Conversely, the *Pomacea* group exhibited a substantially higher number of enriched genes and GO terms, with a total of 484 terms (BP: 158, CC: 83, MF: 243) made up of 389 DEPs (**Fig 3A**). Of those, 74 DEPS were upregulated and 315 were downregulated. When we compared the top GO terms for each group, for the *Lissachatina* group we found translation (13 proteins) was the most enriched term, followed by cytoplasm (12), structural constituent of ribosome (11), and ATP binding (10). In contrast, the highest terms for the *Pomacea* group were ATP binding (60), followed by cytoplasm (44), translation (39), and structural constituent of ribosome (39; **Fig 3A**). This disparity suggests that the *Pomacea* group displays greater molecular and cellular activity, potentially reflecting a broader metabolic and physiological response to infection compared to the *Lissachatina* group. Moreover, the pronounced difference in the number of downregulated DEPs for both groups, particularly within molecular function, suggests the widespread modulation of enzymatic activity, binding, and other molecular mechanisms.

Next, a top five GO enrichment terms per ontology analysis revealed distinct transcriptional responses to infection between the *Lissachatina* and *Pomacea* groups. In the *Lissachatina group*, most of the enriched terms were downregulated, with the biological process category showing the highest enrichment. Key downregulated terms included sulfur compound metabolic process, ribosomal small subunit assembly, and GMP biosynthetic process, while arginine biosynthetic process via ornithine was the only upregulated term. The cellular component and molecular function categories had the next highest terms with membrane coat (downregulated) and oxygen carrier activity (upregulated), respectively (**Fig 3b**). In contrast, all of the top-enriched GO terms in the *Pomacea* group were down-regulated across all three ontologies. The top terms were equally enriched, with regulation of protein catabolic process, cytosolic large ribosomal subunit, and proteasome-activating activity representing the most significant terms within the biological process, cellular component, and molecular function categories, respectively (**Fig 3b**). This uniform down-regulation in the *Pomacea* group suggests a broad repression of cellular functions, whereas the *Lissachatina* group retains some selective activation of stress and metabolic pathways. Furthermore, a chord analysis of the two groups showed a total of 31 DEPs were identified in the *Lissachatina* group, including 9 upregulated and 22 downregulated proteins (**S3 Fig**). Conversely, the *Pomacea* group had 65 DEPs, all of which were downregulated (**S3 Fig**).

**Fig 4:**
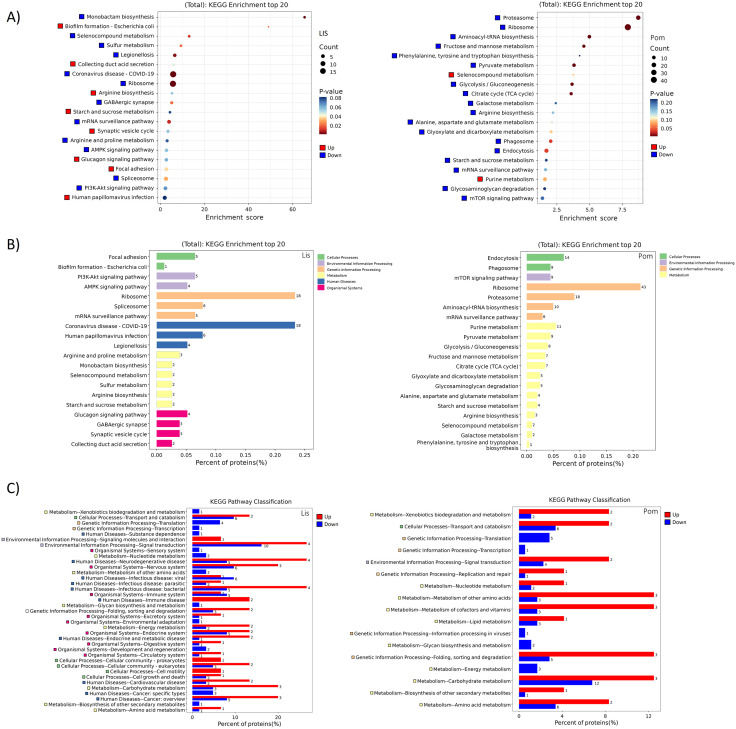
KEGG pathway enrichment analysis of differentially expressed proteins (DEPs) in infected *Lissachatina* (Lis) and *Pomacea* (Pom) groups. (A) Bubble plot of the top 20 most significantly enriched KEGG pathways (Level 3 classification) based on p-values (-log₁₀). Bubble size represents gene count, and color scale denotes significance (p-value). (B) Bar graph summarizing the top 20 KEGG categories (Level 2) derived from the annotated pathways. **(C)** Bar chart illustrating the number of upregulated and downregulated DEPs within each Level 2 KEGG category. Note: Full details of GO terms can be found in S1 Dataset.

Next, a Level 3 KEGG pathway enrichment analysis was performed to cluster DEPs into orthologous groups (KOs) and map them to known metabolic and cellular pathways (Fig 4). In the *Lissachatina* group, the majority of the top 20 enriched pathways (12 out of 20) were significantly downregulated. Notably, pathways associated with detoxification and redox balance, including monobactam biosynthesis, sulfur metabolism, and selenocompound metabolism, were suppressed, suggesting reduced antioxidant activity and impaired oxidative stress defences (**Fig 4A**). Additionally, PI3K-Akt signaling and ribosome-related pathways were downregulated, indicating decreased cell growth, weakened immune signaling, and repression of protein synthesis (**Fig 4A**). Interestingly, several pathways annotated in KEGG under “human disease” (e.g., coronavirus infection, legionellosis) were also significantly downregulated. Although these labels originate from KEGG’s human-based classification framework, they represent conserved cellular functions such as cytoskeletal organization, vesicular trafficking, and protein degradation, which are broadly shared across metazoans [33]. The proteins contributing to these terms (e.g., tubulin β-chain, proteasome α-subunit, V-type proton ATPase, GAPDH, α-actinin, Rab GTPases, and serine/threonine phosphatases) are core regulators of stress response and intracellular transport in invertebrates. In this context, the downregulation of these human disease pathways in *Lissachatina* snails likely represents suppression of analogous stress and immune-related responses rather than direct involvement in human disease. Conversely, upregulated pathways included biofilm formation, collecting duct acid secretion, and arginine biosynthesis (a precursor to nitric oxide). These may reflect compensatory stress and immune responses, as proteins in these pathways are linked to cytoskeletal organization, pH regulation, and nitric oxide-mediated antimicrobial activity, respectively. When placed into Level 2 KEGG categories, we found there was a predominance of pathways associated with human disease (e.g., viral, bacterial, and parasitic infections), comprising 59 proteins across nine KO terms (**Fig 4B**). Additionally, pathways related to genetic information processing (e.g., ribosome, spliceosome, and mRNA surveillance) were enriched, comprising 34 proteins in four KO terms. Furthermore, when classified, we found these categories were largely driven by downregulated proteins, with 34 downregulated and 27 upregulated genes in human disease pathways, and 36 downregulated and 2 upregulated in genetic information processing (**Fig 4C**). The predominance of “human disease” and “genetic information” categories reflect annotation overlap with conserved eukaryotic defence and signalling mechanisms, rather than true pathogenic pathways.

When looking at the Level 3 KEGG pathway enrichment of the *Pomacea* group, we found the majority of enriched pathways (18 of 20) were significantly downregulated (**Fig 4A**). The top ranked downregulated pathways were proteasome, ribosome, and aminoacyl-tRNA biosynthesis, indicating a potential disruption of protein synthesis and degradation. This widespread repression suggests that *A. cantonensis* infection interferes with the host’s proteostatic machinery, potentially impairing hemocyte activity and tissue repair. Other top pathways were metabolic in nature, with fructose and mannose metabolism, pyruvate metabolism, glycolysis/gluconeogenesis, and citrate cycle (TCA) being downregulated, suggesting infection may be reducing host metabolic efficiency. Similarly, the downregulation of mRNA surveillance, mTOR signaling pathways, phagosome formation and endocytosis were observed, implying a decrease in cellular stress response, growth regulation and compromised immune function. Together, these patterns reflect parasite-driven suppression of metabolic and immune functions, consistent with the downregulated pathways observed in the *Lissachatina* group. Only two pathways, selenocompound metabolism and purine metabolism, were upregulated, possible as a result of oxidative stress mitigation, and DNA/RNA repair, respectively. When grouped into Level 2 categories, we found the *Pomacea* group showed strong enrichment in genetic information processing (e.g., ribosome, proteasome, and aminoacyl-tRNA biosynthesis) pathways, with 77 proteins across four KO terms (Fig. 4B). Additionally, metabolic (e.g., purine metabolism, pyruvate metabolism, and glycolysis) pathways were also enriched, comprising 68 proteins in 13 terms. The majority of these enriched pathways were driven by downregulated proteins for both genetic information processing (92 downregulated and 6 upregulated) and metabolism (53 downregulated and 19 upregulated; **Fig 4C**), further supporting extensive suppression of core biosynthetic and energy-producing functions in *Pomacea* during infection.

### 2.3 Protein–protein association of DEPs

**Fig 5:**
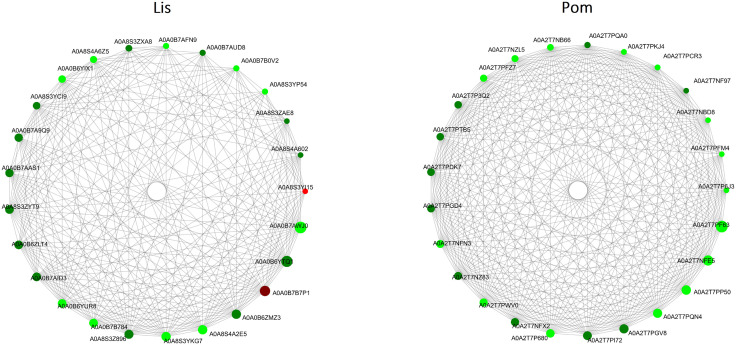
Protein–protein association (PPA) networks of the top 25 differentially expressed proteins (DEPs) in *Lissachatina* (LIS) and *Pomacea* (POM) groups following *A. cantonensis* infection. The PPA networks were generated using STRING and visualized with Cytoscape, highlighting significant DEPs. Each node represents a protein, and each connecting line (edge) represents a predicted functional association between proteins. Node size reflects degree centrality (the number of connections a protein has within the network), indicating its relative importance, while node color denotes log₂ fold change (red = upregulated, green = downregulated). Note: Full details of GO terms can be found in S1 Dataset.

Following GO and KEGG enrichment analyses, a protein–protein association (PPA) network was constructed using the same set of DEPs to explore potential functional relationships among pathways and to identify hub proteins with central regulatory roles. While GO and KEGG enrichment highlight the biological pathways most affected by infection, the PPA network reveals how key proteins are functionally associated within those pathways [32]. In the PPA analysis, each node represents a protein and each edge represents an inferred functional association between two proteins. The degree value indicates how many connections (edges) a protein has within the network, serving as a measure of its centrality. Proteins with higher degree values are considered hub proteins that occupy key regulatory positions, linking multiple biological processes. The PPA network of the *Lissachatina* group revealed nearly all of the top proteins (ranked by degree) were downregulated, except for two, A0A0B7B7P1 (proteasome subunit alpha) and A0A8S3YI15 (tropomyosin), which were upregulated (**Fig 5** and **Table 1**). The most highly connected node was a Tr-type G domain-containing protein, followed by T-complex protein 1 subunit theta, proteasome subunit alpha type, and RNA processing proteins, such as RNA helicase and multiple RRM domain-containing proteins (**Fig 5 and Table 1**). In the *Pomacea* group, the PPA analysis revealed that all of the top proteins were all downregulated. Several highly connected proteins were identified, with two Tr-type G domain-containing proteins being the most connected nodes (**Fig 5 and Table 1**). Additional proteins, such as phosphoglycerate kinase, serine/threonine-protein phosphatase, and several T-complex protein 1 subunits were also highly connected.

**Table 1:** Top ten hub proteins in PPA networks of infected *Lissachatina* and *Pomacea* groups, ranked by degree value (number of associations per protein). Higher degree values indicate proteins with greater network connectivity and potential central regulatory roles. Proteins found in both snail species are shown in bold.

*Lissachatina* (Lis)*			*Pomacea* (Pom)		
Gene	Protein Name	Degree	Gene	Protein Name	Degree
A0A0B7AWJ0	**Tr-type G domain-** **containing protein**	37	A0A2T7PF63	**Tr-type G domain-containing protein**	221
A0A0B6YTQ1	**T-complex protein 1** **subunit theta**	35	A0A2T7NFE5	**Tr-type G domain-containing protein**	208
A0A0B7B7P1	Proteasome subunit alpha	32	A0A2T7PP50	Phosphoglycerate kinase	202
A0A8S3YKG7	RNA helicase	29	A0A2T7PGV8	MPN domain-containing protein	200
A0A8S4A2E5	RRM domain-containing protein	29	A0A2T7PQN4	**T-complex protein 1 subunit epsilon**	200
A0A0B6ZMZ3	60S ribosomal protein L3	29	A0A2T7PI72	Serine/threonine-protein phosphatase	197
A0A8S3Z896	Large ribosomal subunit protein eL6	28	A0A2T7P680	**T-complex protein 1 subunit beta**	195
A0A0B6YUR8	Elongation factor 1-gamma	27	A0A2T7NFX2	**T-complex protein 1 subunit gamma**	193
A0A0B7B784	RRM domain-containing protein	27	A0A2T7PWV0	Small ribosomal subunit protein RACK1	193
A0A0B6ZLT4	Inosine-5’-monophosphate dehydrogenase	27	A0A2T7NFN3	AAA + ATPase domain-containing protein	192

Note: *All *Lissachatina* proteins listed were inferred from homology based on sequence similarity to annotated proteins from *Arion vulgaris* and *Candidula unifasciata*.

When comparing the PPA network analysis of both *Lissachatina* and *Pomacea* snails, we found several highly represented proteins present in both groups. This included Tr-type G domain-containing protein, RNA helicase, T-complex protein 1 subunits (α, β, γ, δ, ε, and θ), proteasome subunits, ribosomal proteins (60S and 40S), elongation factors (EF1-gamma), lysine–tRNA ligase, IMP dehydrogenase, AAA + ATPase domain-containing proteins, translation elongation factors, and eukaryotic peptide chain release factors. However, only Tr-type G domain-containing proteins were present in the top ten for both groups, ranked as number 1 (**Table 1**). This suggests that both groups employ conserved stress-response mechanisms alongside translation regulation and nucleic acid-based defences during *A. cantonensis* infection. This shared network topology suggests that both snail species employ conserved cellular defence mechanisms against *A. cantonensis* infection, dominated by stress-response, proteostasis, and translational regulation processes, possibly reflecting convergent responses to parasitic stress. Identifying these hub proteins can reveal central components of the host’s response to *A. cantonensis* infection and potential molecular targets for further study. However, the *Lissachatina* network exhibited substantially fewer connections (lower node degree values) than the *Pomacea* network. This difference likely reflects the limited number of identified DEPs and the incomplete proteomic annotation available for *Lissachatina fulica*, which constrains STRING-based homology mapping and reduces detectable associations. In contrast, *Pomacea canaliculata* benefits from a well-annotated reference genome, resulting in a denser and more connected network.

## 3. Discussion

The emergence of angiostrongyliasis in new regions presents major challenges in diagnosis, epidemiological tracking, and understanding host-parasite biology. However, despite significant advances in molecular techniques, many aspects of the genetic and molecular pathways involved in host-parasite interactions between *A. cantonensis* and their gastropod hosts remain poorly understood. Our findings reveal distinct proteomic responses in *Lissachatina* and *Pomacea* snails upon *A. cantonensis* infection, shedding light on some of the molecular mechanisms that underpin host susceptibility and parasite transmission. The *Pomacea* group yielded significantly more peptides, proteins, and broader sequence coverage than the *Lissachatina* group. Likewise, a differential expression analysis revealed more DEPs in the *Pomacea* group than the *Lissachatina* group, with both groups showing a predominance of downregulated proteins. This difference is likely due to the lack of gastropod proteomes, with only seven available, specifically *Biomphalaria glabrata* (UP001165740 and UP000076420), *Biomphalaria pfeifferi* (UP001233172), *Elysia chlorotica* (UP000271974)*, Elysia crispata* (UP001283361), *Lymnaea stagnalis* (UP001497497), and *Pomacea canaliculata* (UP000245119). Consequently, protein identification and annotation in *Lissachatina* was less comprehensive.

The GO enrichment showed that most DEPs were related to molecular functions, particularly ATP binding, RNA binding, GTP binding, ATP hydrolysis, and ribosomal structure. Both snail groups exhibited downregulation of unfolded protein binding and ribosomal components, indicating impaired proteostasis and reduced protein synthesis, suggesting a conserved metabolic and transcriptional suppression in response to infection. The *Lissachatina* group also showed decreased RNA binding, while the *Pomacea* group had reduced expression of protein folding chaperones and proteasome-related proteins, implying disrupted protein quality control and endoplasmic reticulum stress. Notably, *Lissachatina* upregulated oxidoreductase and oxygen carrier activity, possibly as a result of oxidative stress mitigation or hypoxia induced by infection. This is consistent with previous studies which have observed that *A. cantonensis* infection induces biochemical disturbances, inflammation, and reduction in reproductive capacity in gastropods [34,35]. At the biological process level, the *Lissachatina* group upregulated the arginine biosynthesis pathway via ornithine, possibly reflecting increased arginase activity, which may impair NO-mediated immune defence. As for the biological process categories, we found the *Lissachatina* group upregulated the arginine biosynthetic pathway via ornithine, which could impair effective anti-parasitic responses, as seen in other Heterobranchia species like *Biomphalaria* [36]. However, other core processes, like GMP biosynthesis, translation, and sulfur metabolism, were suppressed. The *Pomacea* group similarly exhibited broad downregulation across biosynthetic and catabolic pathways. Both snails also showed reduced expression in key cellular components, particularly ribosomal and proteolytic machinery. Altogether, these findings suggest *A. cantonensis* infection induces widespread metabolic and immunological suppression in both intermediate snail hosts.

The KEGG pathway analysis revealed broad suppression of core biosynthetic and stress-response pathways in both snail groups. In the *Lissachatina* group, key metabolic and regulatory pathways, such as ribosome, mRNA surveillance, spliceosome, and PI3K-Akt and AMPK signaling, were downregulated, suggesting a host energy-conservation strategy or parasite-driven metabolic reprogramming. Comparable proteomic suppression of ribosomal and biosynthetic machinery has been reported in *Biomphalaria glabrata* infected with *A. cantonensis* [22,24], supporting the theory of a conserved host response across heterobranch snails involving translational repression and oxidative stress imbalance. However, selective upregulation of pathways like arginine biosynthesis, biofilm formation, and vesicle trafficking indicates targeted compensatory responses, potentially supporting nitric oxide-mediated immunity and cellular signaling. In the *Pomacea* group, nearly all major metabolic pathways, such as the proteasome, glycolysis, TCA cycle, and aminoacyl-tRNA biosynthesis, were downregulated, pointing to extensive metabolic suppression. Only two pathways, selenocompound and purine metabolism, were upregulated, potentially reflecting a response to oxidative stress, and the need for nucleotide synthesis and turnover. Moreover, the protein–protein association analysis further showed significant downregulation of Tr-type GTPases and T-complex chaperonins in both snail groups. These proteins are essential for translation [37] and cytoskeletal protein folding [38], and their loss may impair proteostasis, weaken cytoskeletal integrity, and limit effective immune responses under *A. cantonensis* infection. However, their direct roles in gastropod immunity remain poorly understood. In the *Lissachatina* group, most of the remaining proteins were downregulated, particularly those involved in transcription (e.g., RNA helicases), translation (ribosomal subunits, EF1-γ), and nucleotide biosynthesis (IMPDH), indicating widespread suppression of core biosynthetic functions. Only proteasome subunit and tropomyosin were upregulated, suggesting a limited immune response, as tropomyosin supports hemocyte activation and movement, but without broader protein synthesis, this response may be ineffective. Similarly, proteasome activation could reflect cellular stress or parasite-driven degradation of host proteins. In the *Pomacea* group, all top-ranked proteins were downregulated, such as those related to cytoskeletal organization (T-complex protein 1 subunits), energy metabolism (phosphoglycerate kinase), protein folding and degradation (MPN domain-containing protein, AAA + ATPase), and signal transduction (GTP-binding proteins, RACK1, and serine/threonine-protein phosphatase). This pattern suggests a systemic shutdown of immune and metabolic functions, likely impairing hemocyte mobility, effector production, and cellular energy, further highlighting parasite-mediated suppression in both snail hosts.

Together, our results indicate that both *Lissachatina* and *Pomacea* snails exhibit broadly suppressed immune and metabolic responses during *A. cantonensis* infection. However, it remains unclear whether these effects are unique to *A. cantonensis* or common among helminthic parasites, such as other gastropod-borne metastrongyloidea and trematodes like *Schistosoma*. Notably, *Lissachatina* and *Pomacea* belong to the clades Heterobranchia and Caenogastropoda, respectively, which diverged approximately 400 million years ago [39]. Despite their deep evolutionary separation, both snail species are highly susceptible to *A. cantonensis* and appear to share similar patterns of immune suppression in response to infection. This susceptibility likely reflects the deep evolutionary history of parasitism between gastropods and nematodes, with gastropod-borne parasitism having evolved independently multiple times [40–42]. Furthermore, neither *Lissachatina* and *Pomacea* snails are native to East Asia and therefore lack prior evolutionary adaptation to *A. cantonensis*, unlike some endemic East Asian gastropod species. For example, Lv et al. (2009) [15] found that *A. cantonensis* infections were overwhelmingly concentrated in the invasive snail species *L. fulica* (13.4%) and *P. canaliculata* (6.8%) in their nationwide survey across China, whereas native freshwater (0.05%) and land snail (0.03%) species exhibited near-zero infection prevalence. In contrast, terrestrial slugs showed a moderate infection rate of 6.5%. These findings indicate that the endemicity of *A. cantonensis* in mainland China is primarily attributable to *A. fulica*, *P. canaliculata*, and certain terrestrial slug species. This difference in host compatibility likely enhances the parasite’s environmental persistence and helps explain why invasive snails often act as major amplifiers of *Angiostrongylus* transmission in newly colonized regions [15,43]. However, no proteomic studies on native East Asian snails have yet been conducted to explain why they show lower infection prevalence in the wild.

To our knowledge, this is the first study to investigate the proteomic changes in *Lissachatina* and *Pomacea* snails in response to *A. cantonensis* infection. However, other comparable studies such as Mendes et al. (2020) [24] also show the downregulation of core biosynthetic proteins in *Biomphalaria glabrata* infected by *A. cantonensis*. This suggests a conserved host response or parasite strategy, marked by translational repression and proteostatic stress. There have been several proteomic studies on *Pomacea canaliculata* under chemical stress [44–47]. Boraldi et al. (2021) [48] found *Pomacea canaliculata* infected with malacopathogenic *Phasmarhabditis* nematodes exhibited a strong upregulation of antioxidant enzymes, heat-shock proteins, glycolytic/TCA cycle enzymes, and cytoskeletal remodelers. However, our *Pomacea* data showed the opposite as *Pomacea* infected with *A. cantonensis* had widespread downregulation of these same pathways, suggesting that *A. cantonensis* may uniquely suppress host stress and immune defences to support its development. Moreover, Yue et al. (2023) [49] showed that *Pomacea canaliculata* infected with *A. cantonensis* exhibit significant downregulation of the G-type lysozymes in multiple tissues (e.g., gills, intestine, hepatopancreas, and kidney) across infection stages, suggesting the parasite suppresses key immune responses to enhance its survival in the snail host.

Despite this study providing novel insights into the proteomic responses of *Lissachatina fulica* and *Pomacea canaliculata* to *A. cantonensis* infection, several limitations should be acknowledged. First, the *Lissachatina* samples were obtained from naturally infected snails, whereas *Pomacea* were experimentally infected under controlled laboratory conditions. Although *Lissachatina* were maintained for three weeks prior to dissection and L3 larvae were confirmed microscopically in both snail species, differences in parasite load, infection intensity, and potential host exposure history may still have influenced the observed proteomic patterns. Secondly, the lack of complete gastropod proteomes limited the depth of protein identification, particularly for *Lissachatina*, for which no reference genome is currently available. Consequently, some differentially expressed proteins may remain unannotated or inaccurately classified. Finally, the proteomic analyses were conducted at a single post-infection time point, which restricts temporal interpretation of host responses during different parasite developmental stages.

In conclusion, our results reveal broad immunological and metabolic suppression in both intermediate hosts, highlighting the possible ability of *A. cantonensis* to modulate and dampen the immune responses of its snail hosts. These findings advance our understanding of the transmission biology of *A. cantonensis* and identify possible targets for future control strategies. However, to develop more effective prevention and control measures, a deeper and more comprehensive understanding of the parasite’s life cycle and its mechanisms of host manipulation is essential.

## 4. Materials and methods

### 4.1 Snail collection

Wild *Lissachatina fulica* snails were morphologically identified and collected from Danzhou City, Hainan, China (19°24’29.7“N, 109°35’01.4”E) in November 2022. A total of 65 *Lissachatina* snails, with an average shell length of 4-5cm, were collected and transported to the laboratory for subsequent experiments. Similarly, wild *Pomacea canaliculata* snails were morphologically identified and collected from Kangqiao Ecological Park, Shanghai, China (31°09’14.9”N, 121°37’43.0”E) in August 2022. A total of 120 *Pomacea* snails, averaging 3–4 cm in shell length, were collected and transported to the laboratory for subsequent experiments. Snails were maintained at room temperature under conditions simulating their natural habitats. To ensure that any *Angiostrongylus cantonensis* infections had reached full maturity, the snails were maintained under laboratory conditions for three weeks prior to dissection. This holding period allowed for larval development to the third-stage (L3) phase, minimizing variation due to mixed larval stages.

### 4.2 *Angiostrongylus* infection and hemolymph collection

After the three week holding period, the *Lissachatina* snails had their hemolymph collected and were screened for *A. cantonensis* infection (as described in Jiang et al. 2025 [50]). Of the 65 *Lissachatina* snails tested, 21 were found naturally infected with *A. cantonensis*. For the *Pomacea* snails, 40 of the 120 collected snails were randomly selected and tested for natural *A. cantonensis* infection using the lung microscopy method (as described in Zhao et al. 2024 [51]). However, no *A. cantonensis* infected snails were found. Previous years of routine infection surveillance have shown that *A. cantonensis* is not present in Shanghai. As a result, the remaining collected *Pomacea* snails were instead artificially infected with *A. cantonensis* in a laboratory setting. The procedures for snail maintenance and infection followed the protocol described by Zhao et al. 2024 [51]. In brief, 40 *Pomacea* snails were exposed to 200 *A. cantonensis* L1 larvae for 24hrs and then monitored for 28 days to allow development to the infective L3 stage. Additionally, 40 uninfected snails served as negative control and were not exposed to *A. cantonensis*. After the 28 day period, hemolymph was collected from the *Pomacea* snails, which were then dissected to confirm successful *A. cantonensis* infection.

Prior to hemolymph collection, all snails were thoroughly washed to remove surface debris and contaminants. Residual moisture on the shell surface, particularly around the shell aperture, was carefully dried. In the case of *Lissachatina* snails, hemolymph was collected by inserting a sterile lancet into the heart of the snail. For *Pomacea* snails, hemolymph was collected by inserting a sterile lancet at the edge of the operculum and puncturing into the foot muscle. Hemolymph was slowly released, with 1ml being collected per snail. The hemolymph samples were then centrifuged at 800 x g for 15 minutes at 4°C. Next, 300μL of the resulting supernatant (plasma) was carefully transferred into pre-chilled 2ml cryovial tubes and kept on ice. The hemolymph plasma samples were then flash-frozen in liquid nitrogen and stored at −80°C until use. Following hemolymph extraction, all individuals were tested for *A. cantonensis* infection using the lung microscopy method, and individual hemolymph samples were retroactively labelled as being either infected or uninfected. In total the hemolymph was collected from 16 *A. cantonensis* negative *Lissachatina* snails (Lis-Con), 16 *A. cantonensis* positive *Lissachatina* snails (Lis-Ac), 16 *A. cantonensis* negative *Pomacea* snails (Pom-Con) and 16 *A. cantonensis* positive *Pomacea* snails (Pom-Ac).

### 4.3 Hemolymph protein extraction and preparation

Of the *Lissachatina* and *Pomacea* snails collected, eight replicates (four uninfected and four infected) were prepared for each species, with each replicate consisting of pooled hemolymph from four individuals (S1 Table). Of the four replicates per group, only the best three were chosen for proteomic analysis. Protein extraction, enrichment, and purification and analysis were performed by Shanghai Luming Biotechnology Company using the magnetic nanoparticle suspension method. Briefly, 1mg (40µL) of magnetic nanomaterial was suspended in 100µL of wash buffer, followed by removal of the supernatant. Next, 100µL of hemolymph, pre-mixed with an equal volume of wash buffer, was added and incubated at 37°C for 1 hour on a 360° rotary mixer to facilitate protein binding. Unbound components were removed through multiple wash steps, leaving the enriched proteins retained on the magnetic nanoparticles. For proteolysis, 40µL of Reagent A (reductive alkylation and enzymatic hydrolysis solution) was added to the washed magnetic nanoparticles and thoroughly mixed by pipetting. Next, 1.2µL of Reagent B (reducing agent) was added, and the mixture was heated to 95°C for 5 minutes at 1000 rpm. Once cooled to room temperature, 2µL of Reagent C (equilibration buffer) and 5µL of Reagent D (proteolytic enzyme) were added, thoroughly mixed, and incubated at 37°C for 2 hours. Proteolysis was terminated by adding 3µL of Reagent E (stopping reagent), resulting in the formation of a precipitate. The mixture was centrifuged at 20,000g for 1 minute, and the supernatant was collected for subsequent desalting.

Peptide desalting was performed using a desalting column. The column was first activated with 100µL of methanol and centrifuged at 700g for 1 minute. To remove impurities, 100µL of Conditioning Buffer was added and centrifuged under the same conditions. The column was then equilibrated by adding 100µL of Wash Buffer and centrifuging at 700g for 1 minute. Next, 1–30µg of the sample was then loaded into the column and centrifuged at 700g for 1 minute. This process was repeated to ensure complete loading. The column was then subsequently washed with 100µL of Wash Buffer and centrifuged at 700g for 1 minute, repeating the step for thorough purification. For elution, 30µL of Elution Buffer was added and centrifuged at 700g for 1 minute, with the process repeated to obtain a final sample volume of 60µL. Finally, the eluted peptides were concentrated using a vacuum-frozen centrifugal concentrator in preparation for further analysis.

### 4.4 Proteomic analysis (LC-MS/MS)

The proteomic analysis was performed by Shanghai Luming Biotechnology Company using a 4D label-free quantitative proteomic approach. In brief, liquid chromatography-mass spectrometry (LC-MS) was employed to separate concentrated peptides using a 25 cm C18 analytical column (25 cm × 75μm ID, 1.6μm C18, IonOpticks) at a flow rate of 300nL/min. Peptides were eluted using a binary solvent system consisting of mobile phase A (99.9% H₂O, 0.1% formic acid) and mobile phase B (99.9% acetonitrile, 0.1% formic acid). The gradient elution conditions were as follows: 0 ~ 75 min: 2–22% mobile phase B; 75 ~ 80 min: 22–37% mobile phase B; 80 ~ 85 min: 37–80% mobile phase B; and 85 ~ 90min: 80% mobile phase B. Mass spectrometry (MS) analysis was conducted using an electrospray ionization (ESI) source with a capillary voltage of 1.4kV. The drying gas temperature was set to 180°C, with a flow rate of 3.0L/min. Data acquisition was performed within a mass range of 100-1700m/z, an ion mobility range of 0.6-1.6Vs/cm², and a collision energy range of 20-59eV.

### 4.5 Bioinformatics and data analysis

The bioinformatic analysis was performed by Shanghai Luming Biotechnology Company. In brief, the raw LC-MS/MS data were analyzed using MaxQuant (version 1.6.17.0) with the Andromeda search engine for label-free quantification (LFQ). Protein identification was performed using the UniProt database, with reference proteomes being used for our *Lissachatina* (uniprot-Helicina-216366-2022.12.19.fasta) and *Pomacea* (uniprot-Pomacea-canaliculata-400727-2022.12.19.fasta) datasets. To improve data reliability, a reverse database and a common contaminant database were included for filtering. The search parameters were optimized to minimize false discoveries, setting the false discovery rate (FDR) at 0.01. Trypsin was used as the digestion enzyme, allowing up to two missed cleavages. Carbamidomethylation of cysteine was set as a fixed modification, while methionine oxidation and N-terminal acetylation were considered variable modifications, with label-free quantification was applied to assess protein abundance.

Next, biological process, cellular component, molecular function and Kyoto Encyclopedia of Genes and Genomes (KEGG) were used to classify the Differentially Expressed Proteins (DEPs) by Gene Ontology (GO) enrichment analysis. The GO analysis was done using the Gene Ontology Consortium database, which provides a controlled vocabulary to describe gene and protein functions across different organisms. The KEGG analysis (KEGG database release 91.0) was used to analyze the biological pathways associated the DEPs, the KEGG Orthology (KO) system groups genes with similar functions, and these KO entries map to pathways in the KEGG Pathway database. In the KEGG database, pathways are organized hierarchically across three levels: level 1 represents broad functional classes (e.g., metabolism), level 2 corresponds to specific subcategories within these classes (e.g., carbohydrate metabolism), and level 3 includes the most detailed individual pathways (e.g., glycolysis/gluconeogenesis). The KEGG level 1 pathways were classified into seven major categories: cellular processes, environmental information processing, genetic information processing, human diseases, metabolism, organism systems, and drug development. The p-value representing whether the functional set was significantly enriched in the list of differential proteins was calculated using the hypergeometric distribution test, and the p-value was corrected by the Benjamini & Hochberg multiple test to obtain the FDR [52] (S1 Doc). Protein–protein association (PPA) networks were constructed using the STRING database v12.0 (https://string-db.org/) with a confidence score threshold of 0.4. Only annotated DEPs identified from the proteomic analysis were included. STRING predicts functional associations based on evidence from co-expression, homology, curated databases, and text mining. Networks were visualized in Cytoscape v3.10, with node degree (connectivity) calculated using the Network Analyzer plugin. The top 25 most connected proteins (hub nodes) were displayed. Nodes represent proteins, edges represent predicted functional associations, node color reflects log₂ fold change, and node size corresponds to degree centrality.

## Supporting information

S1 Fig Quality assessment and clustering of trusted protein expression data in A. cantonensis-infected and uninfected Lissachatina (Lis) and Pomacea (Pom) sample groups. (A) Principal component analysis (PCA) of hemolymph proteomic profiles from Lissachatina and Pomacea samples. (B) Hierarchical clustering dendrograms based on Euclidean distances of trusted protein expression levels for each group. (C) Boxplots of normalized trusted protein expression values for each biological replicate. (D) Density plots of normalized protein expression values for each replicate. Note: “Ac” indicates A. cantonensis-infected samples, and “Con” indicates uninfected control samples.
(PNG)

S2 Fig Correlation analysis of the top 50 differentially expressed proteins (DEPs) ranked by p-value for the Lissachatina (Lis) and Pomacea (Pom) groups. Pearson correlation coefficients were calculated to assess the similarity in expression patterns between proteins. Higher coefficients (closer to 1) indicate stronger positive correlations. Color reflects the strength of the correlation, with red representing stronger relationships.
(PNG)

S3 Fig Chord diagram of GO enrichment analysis for Lissachatina (Lis) and Pomacea (Pom) groups. The six most significant GO terms (with ListHits >3 and <50) were selected based on descending P-value (-log₁₀) from the GO enrichment analysis. Proteins are displayed on the left by gene name, with red indicating up-regulation and blue indicating down-regulation. The selected GO terms are displayed on the right. Connecting chords represent associations between individual proteins and specific GO terms.
(PNG)

S1 Table Sample information and peptide concentrations for each snail hemolymph supernatant replicate for the Lissachatina fulica and Pomacea canaliculata groups. (DOCX)

S1 Doc Equipment, reagents, and formulas for the 4D label-free proteomics. (DOCX)

S1 Dataset Raw and processed proteomic data files. (ZIP)
