# Supplementary figures and images for: Proteomic profiles of Lissachatina (Heterobranchia) and Pomacea (Caenogastropoda) snails infected with Angiostrongylus cantonensis using 4D label-free quantitative analysis
Source: PLoS Negl Trop Dis. 2025 Dec 8;19(12):e0013812. doi: 10.1371/journal.pntd.0013812 (PMC12685165; doi:10.1371/journal.pntd.0013812)

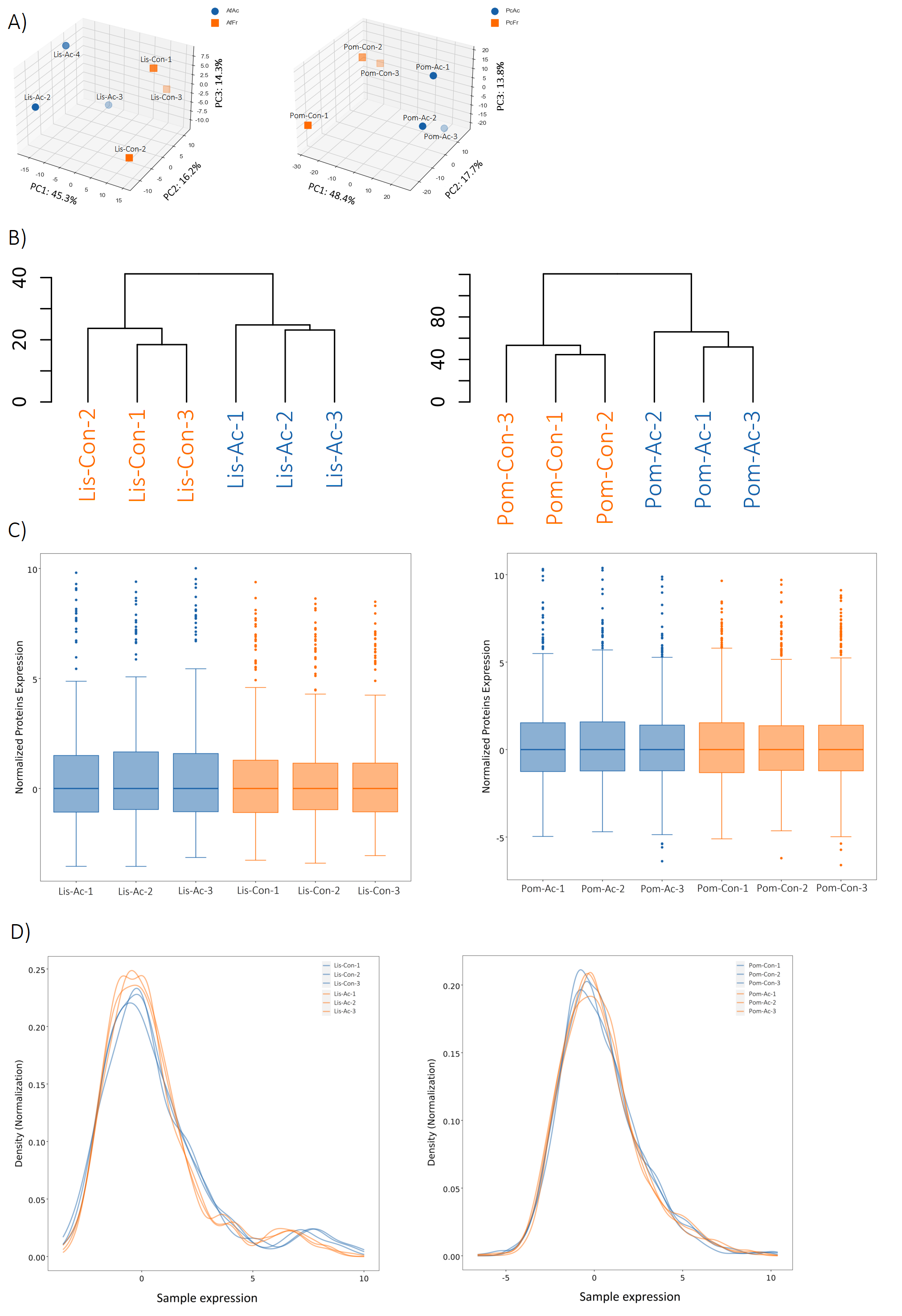

Supplement: S1 Fig — (A) Principal component analysis (PCA) of hemolymph proteomic profiles from Lissachatina and Pomacea samples. (B) Hierarchical clustering dendrograms based on Euclidean distances of trusted protein expression levels for each group. (C) Boxplots of normalized trusted protein expression values for each biological replicate. (D) Density plots of normalized protein expression values for each replicate. Note: “Ac” indicates A. cantonensis-infected samples, and “Con” indicates uninfected control samples. (PNG) [file pntd.0013812.s001.png]

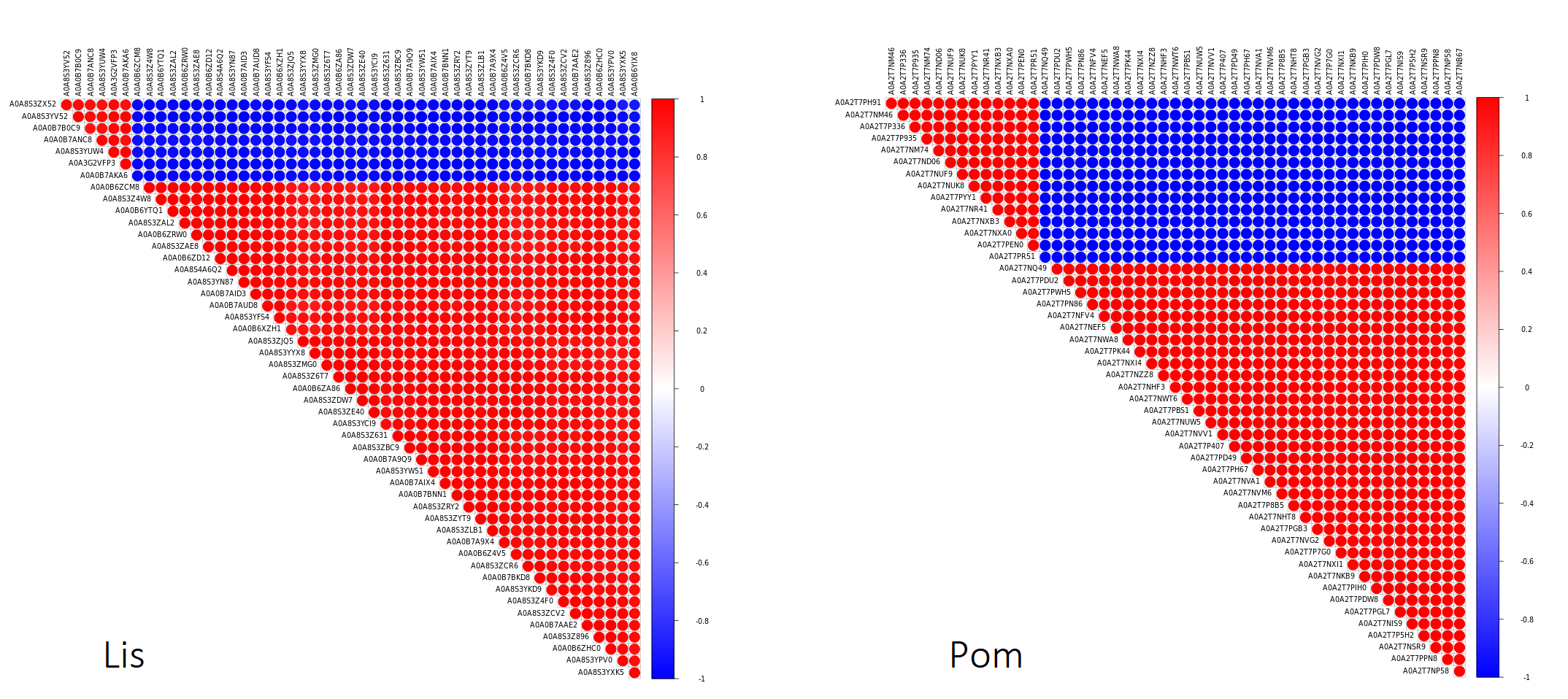

Supplement: S2 Fig — Pearson correlation coefficients were calculated to assess the similarity in expression patterns between proteins. Higher coefficients (closer to 1) indicate stronger positive correlations. Color reflects the strength of the correlation, with red representing stronger relationships. (PNG) [file pntd.0013812.s002.png]

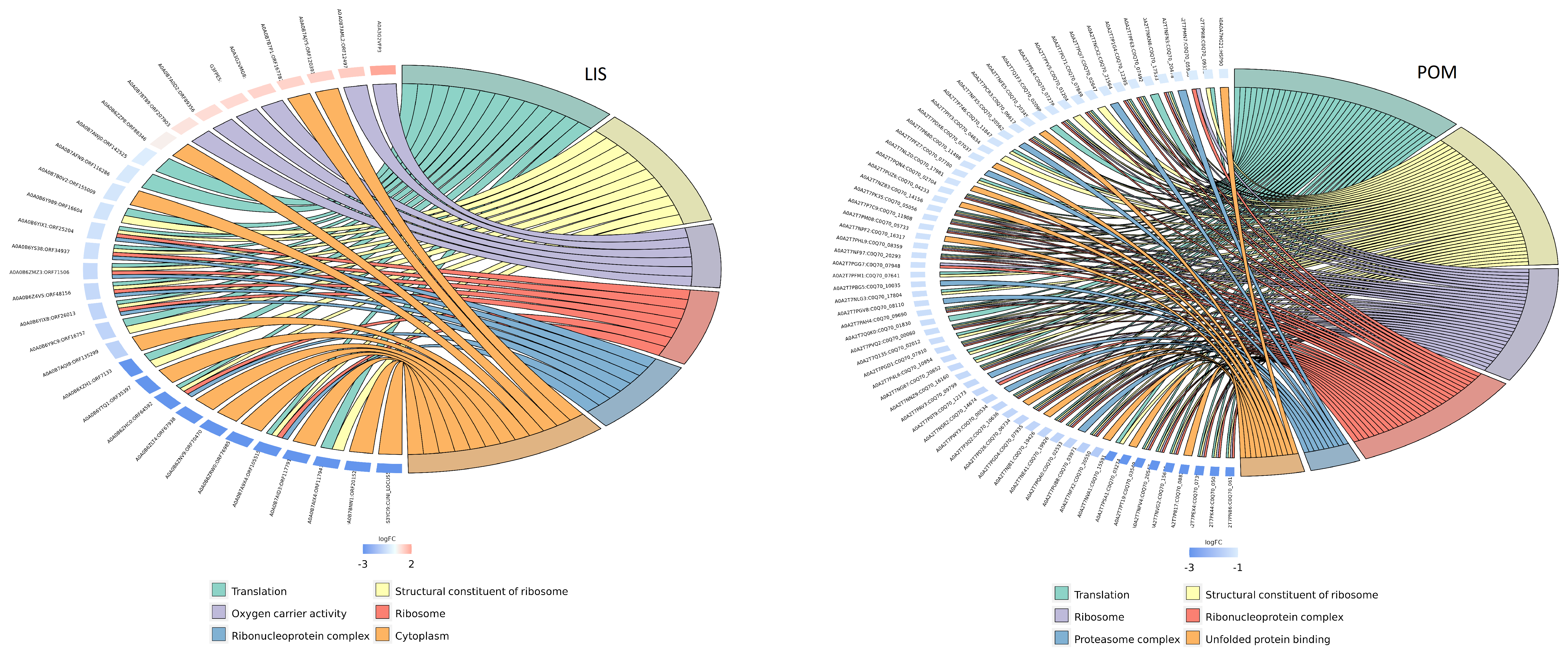

Supplement: S3 Fig — The six most significant GO terms (with ListHits >3 and <50) were selected based on descending P-value (-log₁₀) from the GO enrichment analysis. Proteins are displayed on the left by gene name, with red indicating up-regulation and blue indicating down-regulation. The selected GO terms are displayed on the right. Connecting chords represent associations between individual proteins and specific GO terms. (PNG) [file pntd.0013812.s003.png]
