# Supplementary material for: Proteomic profiles of Lissachatina (Heterobranchia) and Pomacea (Caenogastropoda) snails infected with Angiostrongylus cantonensis using 4D label-free quantitative analysis
Source: PLoS Negl Trop Dis. 2025 Dec 8;19(12):e0013812. doi: 10.1371/journal.pntd.0013812 (PMC12685165; doi:10.1371/journal.pntd.0013812)
Supplement: S1 Table — (DOCX) [file pntd.0013812.s004.docx]

**S1 Table.**  Sample information and peptide concentrations for each snail hemolymph supernatant replicates for the *Lissachatina fulica* and *Pomacea canaliculata* groups.

| *Lissachatina* | | Pooled individuals | Peptide amount | Peptide  concentration |
| --- | --- | --- | --- | --- |
| Uninfected | **Lis-Con-1** | **(n:4)** | **1.2 ug** | **0.06 ug/uL** |
|  | **Lis-Con-2** | **(n:4)** | **1.6 ug** | **0.08 ug/uL** |
|  | **Lis-Con-3** | **(n:4)** | **1.6 ug** | **0.08 ug/uL** |
|  | Lis-Con-4 | (n:4) | 2.2 ug | 0.11 ug/uL |
| Infected | Lis-Ac-1 | (n:4) | 1.4 ug | 0.07 ug/uL |
|  | **Lis-Ac-2** | **(n:4)** | **1.6 ug** | **0.08 ug/uL** |
|  | **Lis-Ac-3** | **(n:4)** | **1.2 ug** | **0.06 ug/uL** |
|  | **Lis-Ac-4** | **(n:4)** | **1.4 ug** | **0.07 ug/uL** |
| *Pomacea* | | Pooled individuals | Peptide amount | Peptide concentration |
| Uninfected | **Pom-Con-1** | **(n:4)** | **2.8 ug** | **0.14 ug/uL** |
|  | **Pom-Con-2** | **(n:4)** | **2.2 ug** | **0.11 ug/uL** |
|  | **Pom-Con-3** | **(n:4)** | **2 ug** | **0.1 ug/uL** |
|  | Pom-Con-4 | (n:4) | 2.4 ug | 0.12 ug/uL |
| Infected | **Pom-Ac-1** | **(n:4)** | **2.6 ug** | **0.13 ug/uL** |
|  | **Pom-Ac-2** | **(n:4)** | **2.8 ug** | **0.14 ug/uL** |
|  | **Pom-Ac-3** | **(n:4)** | **1.8 ug** | **0.09 ug/uL** |
|  | Pom-Ac-4 | (n:4) | 2.2 ug | 0.11 ug/uL |

Note: Sample volume per replicate was 20uL in volume. Highlighted and bold samples were selected for use.
