# Supplementary material for: Proteomic profiles of Lissachatina (Heterobranchia) and Pomacea (Caenogastropoda) snails infected with Angiostrongylus cantonensis using 4D label-free quantitative analysis
Source: PLoS Negl Trop Dis. 2025 Dec 8;19(12):e0013812. doi: 10.1371/journal.pntd.0013812 (PMC12685165; doi:10.1371/journal.pntd.0013812)
Supplement: S1 Doc — (DOCX) [file pntd.0013812.s005.docx]

**S1 Doc**

Instruments:

- TimsTOF Pro2 (Bruker)

- nanoElute liquid chromatography system (Bruker)

- Tabletop refrigerated centrifuge (Shanghai Lu Xiangyi)

- Thermostatic mixer (Shanghai Jingxin Industrial Development Co., Ltd)

- Lyophilizer (Ningbo Xinzhi)

Reagents:

- Mass spectrometry-grade water (Thermo Scientific)

- Acetonitrile (Thermo Scientific)

- Formic acid (CNW)

- Easy Peptide DeeP de-abundant protein enrichment preparation kit (Easy to Calculate)

- Methanol (Labor)

Prepared Buffers:

- Resuspend Buffer (1% TFA)

1 µL trifluoroacetic acid + 100 µL ultrapure water.

- Condition Buffer (70% ACN, 0.2% TFA)

2 µL trifluoroacetic acid + 298µL ultrapure water + 700µL anhydrous acetonitrile.

- Wash Buffer (0.2% TFA)

2 µL trifluoroacetic acid + 998 µL ultrapure water.

- Elution Buffer (90% ACN, 0.2% TFA)

2 µL TFA + 98 µL ultrapure water + 900 µL anhydrous acetonitrile.

The formula for calculating the p-value of the hypergeometric distribution test is given by:


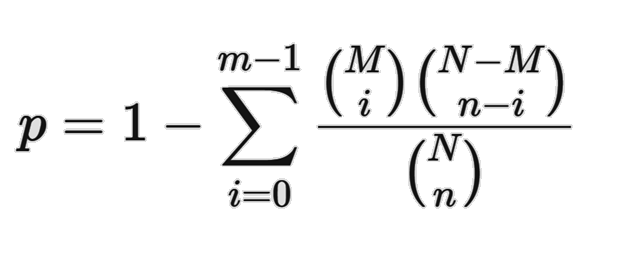


Where:

- *N* is the total number of elements in the population.
- *M* is the total number of elements in the population that are of a specific category.
- *n* is the sample size drawn from the population.
- *m* is the observed number of elements in the sample that belong to the specific category.
- - represents the binomial coefficient, which counts the number of ways to choose *i* elements from *M*.
- The summation accounts for the cumulative probability of observing fewer than *m* elements of the specific category, and the formula calculates the probability of observing at least *m* elements by subtracting this cumulative probability from 1.

The Enrichment Score is calculated using the following formula:


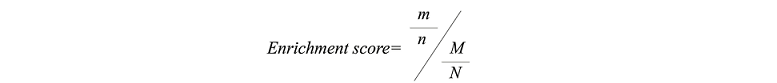


Where:

- *m* is the number of differentially expressed proteins (or genes) in the given functional category.
- *n* is the total number of differentially expressed proteins (or genes).
- *M* is the total number of proteins (or genes) in the given functional category across the background dataset.
- *N* is the total number of proteins (or genes) in the background dataset.
